# Quorum-Sensing C12-HSL Drives Antibiotic Resistance Plasmid Transfer via Membrane Remodeling, Oxidative Stress, and RpoS-RMF Crosstalk

**DOI:** 10.3390/microorganisms13081837

**Published:** 2025-08-06

**Authors:** Yang Yang, Ziyan Wu, Li’e Zhu, Zixin Han, Junpeng Li, Qiaoqiao Fang, Guoqiang Zhu

**Affiliations:** 1 College of Veterinary Medicine, Yangzhou University, Yangzhou 225009, China; mx120220976@stu.yzu.edu.cn (Z.W.); 232001238@stu.yzu.edu.cn (L.Z.); 242001110@stu.yzu.edu.cn (Z.H.); mz120231635@stu.yzu.edu.cn (J.L.); mz120241776@stu.yzu.edu.cn (Q.F.); 2 Jiangsu Co-Innovation Center for Important Animal Infectious Diseases and Zoonoses, Joint Laboratory of International Cooperation on Prevention and Control Technology of Important Animal Diseases and Zoonoses of Jiangsu, Yangzhou 225009, China

**Keywords:** quorum sensing, ribosomal hibernation factor, plasmid conjugation

## Abstract

Antibiotic misuse accelerates resistance dissemination via plasmid conjugation, but quorum sensing (QS) regulatory mechanisms remain undefined. Using *Escherichia coli* (*E. coli*) MG1655 conjugation models (RP4-7/EC600 plasmids), we demonstrate that long-chain acyl-homoserine lactones (C10/C12-HSL) enhance transfer frequency by up to 7.7-fold (200 μM C12-HSL; *p* < 0.001), while quorum-quenching by sub-inhibitory vanillin suppressed this effect by 95% (*p* < 0.0001). C12-HSL compromised membrane integrity via *ompF* upregulation (4-fold; *p* < 0.01) and conjugative pore assembly (*trbBp* upregulated by 1.38-fold; *p* < 0.05), coinciding with ROS accumulation (1.5-fold; *p* < 0.0001) and SOS response activation (*recA* upregulated by 1.68-fold; *p* < 0.001). Crucially, *rpoS* and *rmf* deletion mutants reduced conjugation by 65.5% and 55.8%, respectively (*p* < 0.001), exhibiting attenuated membrane permeability (≤65.5% reduced NPN influx; *p* < 0.0001), suppressed ROS (≤54% downregulated; *p* < 0.0001), and abolished transcriptional induction of conjugation/stress genes. Reciprocal RpoS–RMF (ribosomal hibernation factor) crosstalk was essential for AHL responsiveness, with deletions mutually suppressing expression (≤65.9% downregulated; *p* < 0.05). We establish a hierarchical mechanism wherein long-chain AHLs drive resistance dissemination through integrated membrane restructuring, stress adaptation, and RpoS–RMF-mediated genetic plasticity, positioning QS signaling as a viable target for curbing resistance spread.

## 1. Introduction

The escalating crisis of antibiotic resistance constitutes a critical global public health threat, fundamentally compromising modern therapeutic approaches [1,2]. The rampant overuse and misuse of antibiotics in human medicine (e.g., penicillins, cephalosporins, macrolides, fluoroquinolones) and intensive agriculture (e.g., tetracyclines, colistin, amoxicillin) act as powerful accelerants for the emergence and dissemination of resistant bacteria [3,4]. Crucially, genes conferring resistance (antibiotic resistance genes, ARGs) propagate not only vertically within lineages but also dynamically through horizontal gene transfer (HGT), significantly expanding resistance reservoirs in microbial communities [5,6]. Among HGT mechanisms, bacterial conjugation—the direct cell-to-cell transfer of mobile genetic elements like plasmids—represents a primary vector for the widespread dissemination of ARGs across diverse bacterial populations, thereby exacerbating resistance and complicating treatment strategies [7,8].

Understanding the molecular drivers that regulate the efficiency of conjugative plasmid transfer within this complex landscape is paramount. While antibiotic pressure selects for resistant clones, specific environmental and physiological cues govern the rate of HGT [9]. Bacterial communication via quorum sensing (QS) systems has emerged as a key regulatory node in this process [1,3,10,11,12]. QS enables populations to collectively adapt their behavior in response to cell density through the synthesis, secretion, and perception of diffusible signaling molecules. In Gram-negative bacteria, acyl-homoserine lactones (AHLs) serve as the characteristic QS signals. Growing evidence indicates that specific AHLs significantly influence virulence, biofilm development, and, notably, plasmid conjugation efficiency in various species [4,6,13,14]. However, a critical knowledge gap persists: the precise molecular mechanisms by which AHL signaling orchestrates this enhancement of conjugative transfer—particularly the integrated interplay between membrane dynamics, cellular stress responses, and regulatory networks—remain incompletely defined. Elucidating this intricate signaling cascade is essential for developing strategies to disrupt ARG dissemination mediated by bacterial communication.

Research demonstrates compelling links between AHL-mediated QS and factors governing conjugation efficiency [1,10,15,16]. Evidence indicates that AHL signaling can significantly influence membrane architecture. For instance, investigations into QS inhibition have shown that specific compounds like essential oils of *Origanum compactum* [17,18,19] disrupt QS phenotypes in various bacteria partly by impairing cell membrane integrity and increasing permeability, altering critical barrier functions. This aligns with indications that AHLs themselves may modulate outer membrane permeability, potentially through regulation of porin expression (e.g., OmpF/C), thereby impacting critical steps like donor–recipient contact and DNA passage during conjugation. Concurrently, exposure to certain AHLs has been mechanistically linked to the induction of oxidative stress, resulting in reactive oxygen species (ROS) accumulation. Studies across diverse bacterial systems reveal that QS components and the receptor LsrR play crucial regulatory roles in the oxidative stress response. For example, LsrR was found to bind directly to and repress promoters of key oxidative defense genes (*ahpCF*, *katG*) in *E. coli* [20], highlighting an intimate connection between QS perception and ROS metabolism. ROS accumulation serves a dual role: beyond causing damage, they function as signaling molecules, triggering SOS DNA repair responses and promoting genetic plasticity—phenomena strongly correlated with elevated HGT rates, including conjugation [21]. Furthermore, bacterial stress adaptation pathways, governed by master regulators like RpoS (the stationary phase/stress sigma factor), are indispensable for survival under diverse stresses, potentially creating a cellular environment conducive to plasmid acquisition [22]. Importantly, the intersection of QS and stress adaptation is underscored by observations that environmental stresses, like high salt concentrations [23] or antibiotic pressure (e.g., oxytetracycline) [24], actively induce AHL-mediated QS signaling. This heightened QS activity coordinates community responses, including increased production of extracellular polymeric substances (EPS) and modulation of genes involved in stress tolerance and resistance gene proliferation. The emerging picture suggests QS acts as a sensory hub, integrating environmental cues and orchestrating responses involving membrane dynamics, ROS generation, and stress adaptation networks, all of which can influence the molecular landscape for efficient conjugative transfer.

Observations suggest correlations between AHL signaling and RpoS induction [25,26,27,28]. Additionally, the ribosome modulation factor Rmf, which is expressed during stress (e.g., nutrient limitation) to promote ribosome hibernation (dormancy through 100S dimer formation), has also exhibited potential expression links to RpoS and QS responses in some contexts [29,30,31,32]. Preliminary findings imply that these pathways may converge to foster conditions optimal for plasmid transfer. Nevertheless, a comprehensive mechanistic model detailing how specific long-chain AHLs like C12-HSL—upon cellular perception—induce membrane remodeling, orchestrate oxidative stress and SOS pathway activation, and critically engage in crosstalk between the key stress regulators RpoS and Rmf to ultimately amplify conjugation remains elusive. Fundamental questions persist: Does C12-HSL directly compromise membrane integrity? Is ROS generation a causal factor or an indirect consequence? And most importantly, are RpoS and Rmf functionally interdependent and essential components within the AHL conjugation signal transduction pathway? Resolving these questions is crucial for understanding the physiological underpinnings of QS-mediated resistance spread.

This study elucidates a hierarchical molecular mechanism driving the C12-HSL-mediated enhancement of conjugative plasmid transfer. Using defined *E. coli* MG1655 conjugation models, we demonstrate that C12-HSL markedly increases transfer frequency through concerted mechanisms: inducing membrane restructuring (heightened permeability via OmpF upregulation), provoking significant oxidative stress (ROS accumulation), and activating the SOS DNA repair response. Critically, we identify an essential reciprocal crosstalk between the stress master regulator RpoS and the ribosome hibernation factor Rmf as the core signaling hub for cellular responses to C12-HSL. Genetic deletion of either *rpoS* or *rmf* severely attenuates AHL-induced conjugation, membrane destabilization, and stress responses, unequivocally establishing their non-redundant roles. Our findings delineate a pathway where C12-HSL signaling, transduced through integrated membrane perturbation, stress adaptation, and RpoS–Rmf-mediated genetic and physiological plasticity, culminates in a dramatic increase in ARG dissemination via conjugation, thereby positioning critical QS components as viable targets for mitigating resistance spread.

## 2. Materials and Methods

### 2.1. Bacterial Strains and Culture Conditions

All experiments utilized *E. coli* K-12 strain MG1655 and its derivatives. The conjugation donor strain was MG1655(RP4-7), harboring the broad-host-range plasmid RP4-7 (confers resistance to chloramphenicol [68 μg/mL] and ampicillin [100 μg/mL]). The recipient strain, MG1655(EC600), carried plasmid EC600 (confers rifampicin resistance [300 μg/mL]) [21]. Isogenic deletion mutants (Δ*rmf*, Δ*rpoS*) and complemented strains (Δ*rmf/prmf*, Δ*rpoS/prpoS*) were constructed in this study. Bacteria were routinely cultured at 37 °C in Lysogeny Broth (LB; Haibo Biotechnology, Qingdao, China) with shaking (200 rpm). Solid media contained 1.5% agar (Haibo Biotechnology) supplemented with antibiotics as required. Mueller Hinton (MH) broth (Haibo Biotechnology) was used for minimum inhibitory concentration (MIC) assays. The strains used in this study are listed in Table S1.

### 2.2. Reagents and Quorum Sensing Modulators

The acyl-homoserine lactones (AHLs) N-Hexanoyl-(C6-HSL), N-Octanoyl-(C8-HSL), N-Decanoyl-(C10-HSL), and N-Dodecanoyl-L-homoserine lactone (C12-HSL) (Macklin Biochemical, Shanghai, China, ≥98% purity) were dissolved in dimethyl sulfoxide (DMSO; Solarbio, Beijing, China) to 100 mM stock concentrations. Working solutions (0–200 μM) were freshly prepared in sterile phosphate-buffered saline (PBS; pH 7.2, Solarbio). The quorum sensing inhibitor vanillin (Yuanye Bio, Shanghai, China) was dissolved in DMSO (500 mM stock) [33,34]. The fluorescent probes propidium iodide (PI), *N*-phenyl-1-naphthylamine (NPN), and 2′,7′-dichlorodihydrofluorescein diacetate (DCFH-DA) were obtained from Beyotime Biotechnology (Shanghai, China) and prepared according to manufacturer protocols.

### 2.3. Minimum Inhibitory Concentration (MIC) Determination

The MIC of vanillin against donor and recipient strains was determined by broth microdilution following CLSI guideline M07-A10. Bacterial suspensions adjusted to OD_600_ = 0.5 in MH broth were diluted 1:100, and 100 μL aliquots were dispensed into 96-well microtiter plates [21]. Vanillin was serially diluted two-fold in MH broth (final concentrations: 0.039–5 mM). Wells containing bacteria without vanillin served as growth controls; sterile broth served as a negative control. After 18 h of static incubation at 37 °C, the MIC was defined as the lowest concentration that completely inhibited visible growth. Sub-inhibitory concentrations (1/16–1/2 MIC) were confirmed not to alter growth kinetics through hourly OD_600_ monitoring during shaking incubation (200 rpm, 37 °C).

### 2.4. Plasmid Conjugation Assay

Strains were streaked onto selective agar plates and incubated overnight (16 h, 37 °C). Single colonies were inoculated into 2 mL of antibiotic-supplemented LB broth and grown to mid-log phase (OD_600_ ≈ 0.6; 6 h, 37 °C, 200 rpm). Cells were harvested by centrifugation (8000× *g*, 5 min, 4 °C), washed thrice in sterile PBS to remove residual antibiotics, and resuspended in PBS to a final OD_600_ of 1.0 (≈1 × 10^9^ CFU/mL) for conjugation. Donor and recipient suspensions were combined at a 1:1 volumetric ratio in 1.5 mL microcentrifuge tubes. AHLs were added to final concentrations of 0, 10, 100, or 200 μM. For inhibition assays, vanillin was added to sub-MIC concentrations (0.078–0.625 mM). Mixtures were incubated statically for 16 h at 37 °C. Post-incubation cultures underwent serial decimal dilution in sterile PBS. Transconjugants were enumerated via spot-plating (10 μL) or spread-plating (100 μL) onto LB agar containing rifampicin (300 μg/mL), ampicillin (100 μg/mL), and chloramphenicol (68 μg/mL). Recipient counts were determined on rifampicin-only agar. The conjugation frequency was calculated as (Transconjugant CFU/mL)/(Recipient CFU/mL). Three biological replicates were performed per condition [21].

### 2.5. Scanning Electron Microscopy (SEM)

Bacterial pellets were fixed in 2.5% glutaraldehyde (Solarbio) in PBS (4 °C, 24 h), washed three times with PBS, and post-fixed in 1% osmium tetroxide (1 h). Samples were dehydrated through a graded ethanol series (30%, 50%, 70%, 80%, 90%, and 100%; 15 min per step), followed by critical-point drying using a Leica CPD300 (Leica Microsystems GmbH, Wetzlar, Germany) [21,35]. Dried samples were sputter-coated with gold–palladium (5 nm) and imaged at 5 kV acceleration voltage using a Zeiss GeminiSEM 300 field-emission scanning electron microscope (Carl Zeiss AG, Oberkochen, Germany).

### 2.6. Membrane Integrity Assessment

Inner membrane permeability: Washed cell suspensions (10^7^ CFU/mL in PBS) were incubated with propidium iodide (PI; 0.5 μM final concentration) for 30 min at 37 °C in the dark. After treatment with 200 μM C12-HSL (1 h, 37 °C), fluorescence intensity (excitation: 535 nm, emission: 615 nm) was quantified using a BioTek Synergy H1 microplate reader (Agilent Technologies, Santa Clara, CA, USA). Outer membrane permeability: Cell suspensions were incubated with N-phenyl-1-naphthylamine (NPN; 10 μM final concentration) for 30 min (37 °C, dark). Following C12-HSL treatment (200 μM, 1 h), fluorescence (excitation: 350 nm, emission: 420 nm) was measured as above [21]. Data were normalized to untreated controls.

### 2.7. Intracellular Reactive Oxygen Species (ROS) Measurement

Intracellular ROS levels were quantified using DCFH-DA [21]. Washed cells were incubated with 10 μM DCFH-DA for 30 min (37 °C, dark), washed twice with PBS to remove unincorporated dye, and treated with 200 μM C12-HSL for 1 h (37 °C, dark). Fluorescence (excitation: 488 nm, emission: 525 nm) was measured. A positive control group treated with ROSup (Beyotime) was included. Data were expressed as fold-change relative to untreated cells.

### 2.8. Construction of Isogenic Deletion Mutants and Complementation

Primers used are listed in Supplementary Table S2. Purified PCR products were electroporated (1.8 kV, 200 Ω, 25 μF) into MG1655 harboring the temperature-sensitive plasmid pKD46 (induced with 30 mM L-arabinose) [36]. Transformants were selected on LB agar containing chloramphenicol (30 μg/mL) at 30 °C. Successful chromosomal integration (Δ*rmf::cat*, Δ*rpoS::cat*) was verified by PCR. Plasmid pCP20 (encoding FLP recombinase) was transformed into primary mutants and induced at 42 °C to excise the *cat* cassette. Chloramphenicol-sensitive clones (Δ*rmf*, Δ*rpoS*) were confirmed by PCR and Sanger sequencing (Figure S1).

For genetic complementation, full-length *rmf* or *rpoS* genes were PCR-amplified, cloned into plasmid pBR322 (Takara, Dalian, China) using restriction sites (BamHI/SalI for *rmf*, NdeI/BamHI for *rpoS*), and transformed into respective deletion mutants to generate complemented strains (Δ*rmf/prmf*, Δ*rpoS/prpoS*) [37].

### 2.9. Quantitative Reverse Transcription PCR (qRT-PCR)

Total RNA was isolated from mid-log-phase cultures (OD_600_ = 0.6) ± 200 μM C12-HSL using Vazyme VoZol Reagent. Residual DNA was removed with DNase I (Vazyme, Nanjing, China). RNA purity (A_260/280_ > 1.9) and concentration were determined via a NanoDrop 2000 (Thermo Fisher Scientific Inc., Waltham, MA, USA). First-strand cDNA was synthesized from 1 μg total RNA using HiScript III RT SuperMix with gDNA wiper (Vazyme, Nanjing, China) following manufacturer instructions. Reactions employed SupRealQ Purple Universal SYBR qPCR Master Mix (U+) (Vazyme) on a Roche Lightcycler96 system [38].

### 2.10. Growth Curve Analysis

Overnight cultures were diluted 1:100 in fresh LB broth (±antibiotics). Aliquots (100 μL) were collected hourly, and OD_600_ was measured using a BioTek Epoch microplate spectrophotometer (Agilent Technologies, Santa Clara, CA, USA) [39]. Growth kinetics were compared across strains to normalize conjugation data to biomass.

### 2.11. Statistical Analysis

All experiments were performed with three independent biological replicates. Data are presented as mean ± standard deviation (SD). Unpaired two-tailed Student’s *t*-tests (two-group comparisons) or one-way ANOVA with Tukey’s post hoc test (≥3 groups) were conducted using GraphPad Prism 9.5. Statistical significance thresholds were set at *p* < 0.05, *p* < 0.01, *p* < 0.001, and *p* < 0.0001.

## 3. Results

### 3.1. Ensuring Strain Growth Uniformity and Quantifying AHL-Specific Enhancement of Plasmid Conjugation in E. coli

**Figure 1:**
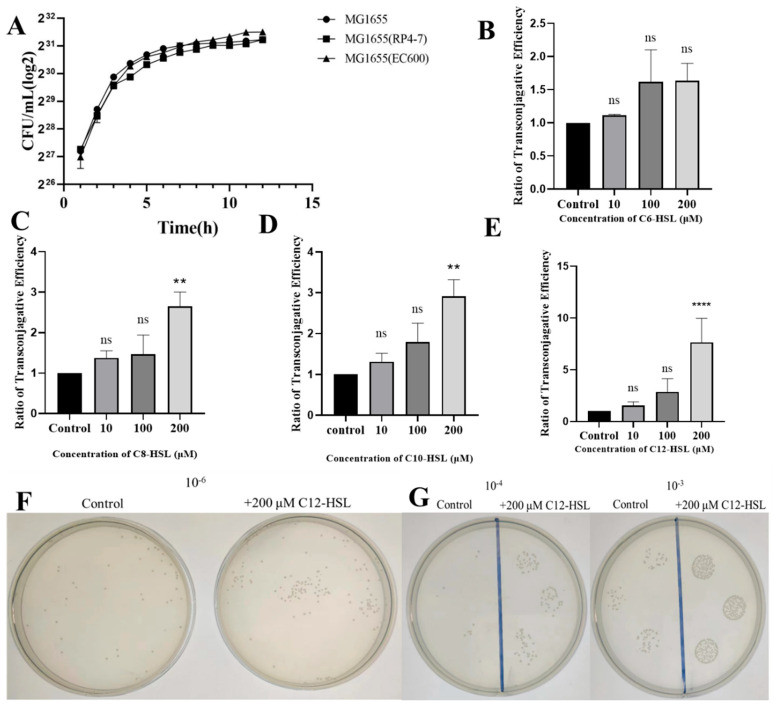
Impact of AHLs on conjugative plasmid transfer in *E. coli*. (**A**) Growth curves of wild-type *E. coli* MG1655, donor MG1655(RP4-7), and recipient MG1655(EC600) strains. OD_600_ measurements were recorded hourly in LB broth to verify uniform growth kinetics prerequisite for conjugation experiments. (**B**–**E**) Conjugation efficiency, quantified as the transconjugant-to-recipient ratio via plate counting, following exposure to (**B**) C6-HSL, (**C**) C8-HSL, (**D**) C10-HSL, or (**E**) C12-HSL at concentrations of 0, 10, 100, or 200 μM. Values denote means ± SD (*n* = 3 biological replicates). Significance markers: ns (non-significant), ** *p* < 0.01, **** *p* < 0.0001 (compared to untreated controls; two-tailed Student’s *t*-test). (**F**) Visualization of transconjugant colonies via plate-spreading assays on rifampicin/ampicillin/chloramphenicol agar after treatment with 200 μM C12-HSL versus control. Bacterial suspensions were plated at a 10^−6^ dilution. (**G**) Spot-plating assays of transconjugants under identical conditions as (**F**) at 10^−3^ and 10^−4^ dilutions.

To establish consistent growth kinetics for donor and recipient strains prior to plasmid conjugation assays, growth curve analyses were conducted on the wild-type *E. coli* strain MG1655, donor MG1655(RP4-7), and recipient MG1655(EC600). All strains exhibited analogous growth kinetics, characterized by time-dependent increases, followed by plateaus, confirming equivalent growth rates without statistical disparities (Figure 1A). Next, we investigated the influence of acyl-homoserine lactones (AHLs) on conjugative plasmid transfer efficiency by measuring transconjugant-to-recipient ratios across concentrations (0, 10, 100, 200 μM). While C6-HSL exposure yielded no significant changes relative to controls (ratio = 1; *p* > 0.05), C8-HSL, C10-HSL, and C12-HSL at 200 μM significantly elevated ratios (3.2, 3.2-adjusted, and 7.7, respectively; all *p* < 0.05 for C8/C10, *p* < 0.001 for C12). Lower concentrations of these AHLs showed no notable effects (Figure 1B–E). The concentration-dependent enhancement for C12-HSL was visually substantiated by intensified colony growth in plate dilution and spot-plating assays, reinforcing the quantitative efficacy observed at 200 μM (Figure 1F,G).

### 3.2. Vanillin Interferes with C12-HSL-Stimulated Plasmid Conjugation in E. coli

**Figure 2:**
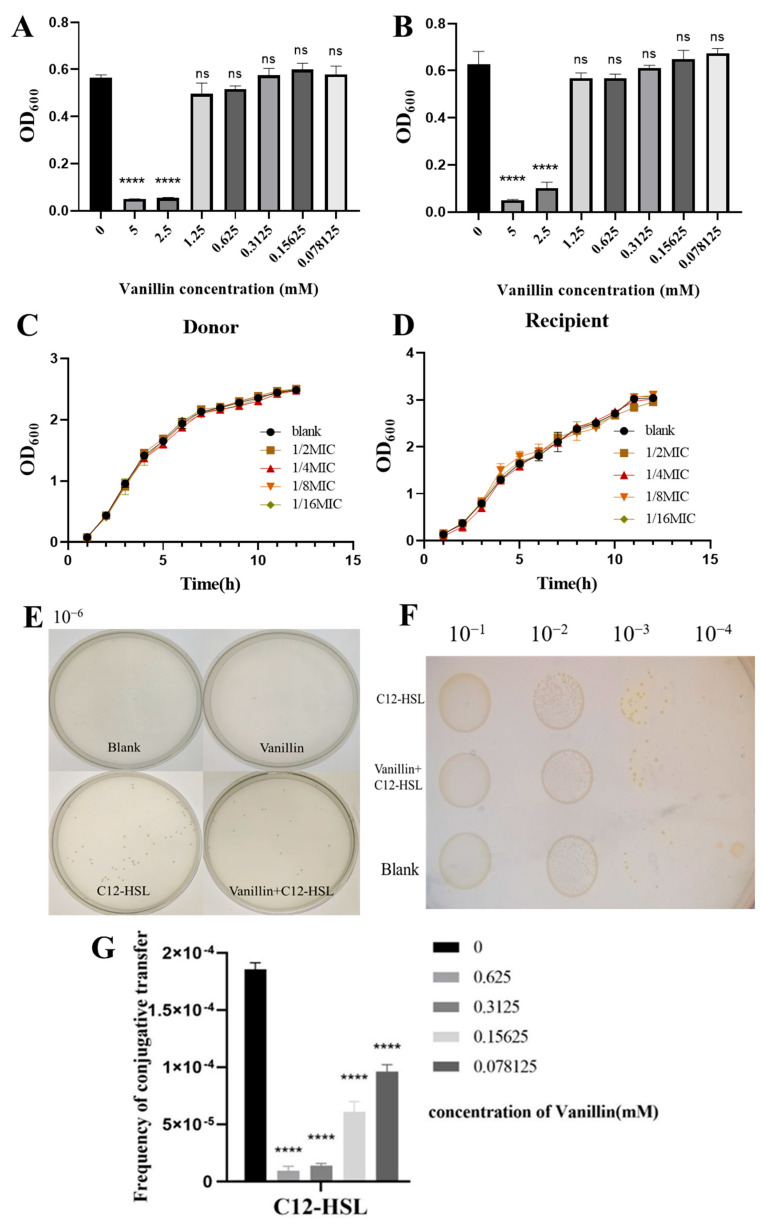
Quorum-sensing inhibition by vanillin abrogates C12-HSL-potentiated conjugative transfer in *E. coli*. (**A**,**B**) Minimum inhibitory concentration (MIC) of vanillin against (**A**) donor MG1655(RP4-7) and (**B**) recipient MG1655(EC600) strains, determined by microdilution broth assay. OD_600_ values (24 h) shown as mean ± SD (*n* = 3). Statistical significance: **** *p* < 0.0001 versus untreated control (0 mM); ns, not significant. MIC defined at 1.25 mM for both strains. (**C**,**D**) Growth kinetics of (**C**) donor and (**D**) recipient strains cultured with sub-MIC vanillin (0.078–0.625 mM). OD_600_ measurements recorded hourly for 15 h demonstrate kinetics indistinguishable from untreated controls (*p* > 0.05, two-way ANOVA). (**E**) Representative plate-spreading assay of transconjugants on triple-antibiotic agar (rifampicin/ampicillin/chloramphenicol). Bacterial suspensions (10^−6^ dilution) plated after treatments: Control (Blank), 0.625 mM vanillin (V), 200 μM C12-HSL (C12), or vanillin + C12-HSL (V + C12). (**F**) Spot-plating assays comparing identical treatments at 10^−3^ and 10^−4^ dilutions. (**G**) Dose-dependent suppression of C12-HSL-enhanced conjugation by vanillin. Conjugation frequencies quantified with 200 μM C12-HSL and increasing sub-MIC vanillin (0–0.625 mM). Values denote mean ± SD (*n* = 3 biological replicates). **** *p* < 0.0001 versus C12-HSL without vanillin (one-way ANOVA with Tukey’s test).

We first determined the minimum inhibitory concentration (MIC) of the quorum sensing inhibitor vanillin for donor [*E. coli* MG1655(RP4-7)] and recipient [MG1655(EC600)] strains using microbroth dilution assays. Vanillin exhibited an MIC of 1.25 mM for both strains, with concentrations ≥1.25 mM showing no significant growth impairment versus untreated controls (ns), while 5 mM significantly reduced growth (*p* < 0.05; Figure 2A,B). Sub-MIC vanillin (0.078–0.625 mM, 1/16–1/2 MIC) did not alter the growth kinetics of either strain across lag, exponential, or stationary phases (*p* > 0.05; Figure 2C,D), confirming its suitability for subsequent QS inhibition experiments.

Plate-based conjugation assays revealed critical mechanistic insights. Sub-MIC vanillin (0.625 mM) alone preserved baseline conjugation efficiency (*p* > 0.05 vs. control; Figure 2E,F), whereas 200 µM C12-HSL robustly enhanced conjugation (*p* < 0.001). Concomitant vanillin exposure significantly inhibited C12-HSL-mediated hyperconjugation, reducing transconjugant density by ∼50% in spot assays (*p* < 0.001; Figure 2F). Dose-response quantification (Figure 2G) demonstrated that 0.625 mM vanillin reduced C12-HSL-stimulated conjugation frequency from 1.80 × 10^−4^ to 9.2 × 10^−6^ (95% suppression, *p* < 0.0001), approximating baseline levels (2.5 × 10^−5^). Lower concentrations (0.078–0.3125 mM) progressively diminished this inhibition, with 0.3125 mM vanillin reducing transfer frequency to 1.36 × 10^−5^ (92.5% suppression). These findings establish that sub-inhibitory vanillin selectively neutralizes AHL-driven conjugative enhancement without impairing basal transfer activity.

### 3.3. C12-HSL Disrupts Membrane Integrity and Induces Oxidative Stress to Facilitate Conjugation

**Figure 3:**
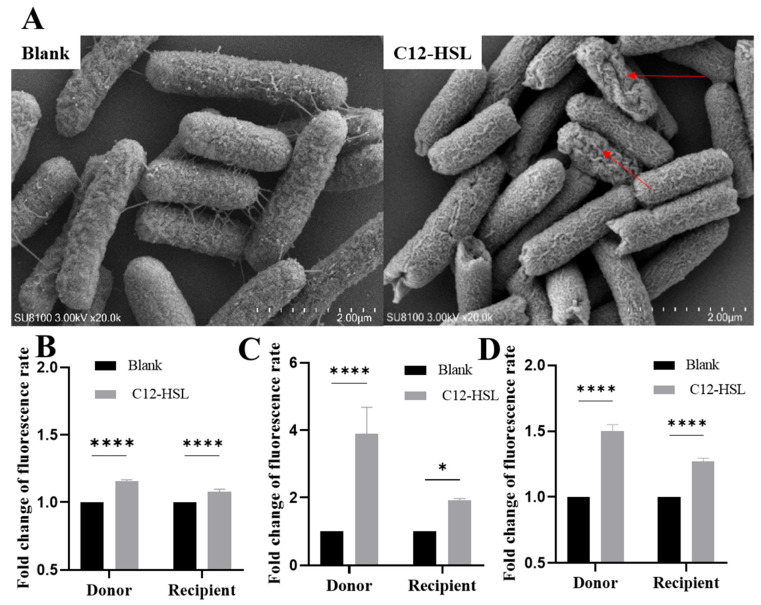
C12-HSL-mediated structural and permeability modifications in *E. coli*. (**A**) Scanning electron microscopy of bacterial surface morphology. *E. coli* cells were analyzed post-incubation with 200 μM C12-HSL for 16 h or under untreated conditions. Representative images display untreated controls (intact, smooth surfaces) and C12-HSL-treated samples (surface disruptions). The red arrow indicates the damaged area on the bacterial surface. (**B**) Quantification of inner membrane integrity via PI fluorometric assay. Bacterial suspensions were exposed to 200 μM C12-HSL for 1 h; PI fluorescence (excitation: 535 nm, emission: 615 nm) was recorded to assess permeability, with data scaled to the untreated baseline. (**C**) Determination of outer membrane permeability employing NPN fluorometry. After a 1 h incubation with 200 μM C12-HSL, fluorescence responses were measured to determine fold changes relative to controls. (**D**) Detection of intracellular ROS using DCFH-DA fluorescence. Cells were treated with 200 μM C12-HSL for 1 h, and ROS levels were gauged at excitation/emission wavelengths of 488/525 nm, presented as fold change over untreated groups. Statistical significance: **** *p* < 0.0001, * *p* < 0.05.

High-resolution SEM imaging exposed critical structural alterations induced by 200 μM C12-HSL in *E. coli*. While untreated bacteria maintained intact envelopes with smooth surfaces (Figure 3A), treated cells exhibited quantifiable ultrastructural damage, including pore formation and membrane breaches (arrows). This physical compromise was biochemically validated through fluorescence-based permeability assays. Propidium iodide staining confirmed a statistically significant 1.5-fold increase in inner membrane permeability for both donor and recipient strains (*p* < 0.0001; Figure 3B), while NPN partitioning revealed substantial outer membrane destabilization (donors: 4-fold increase, *p* < 0.0001; recipients: 2-fold, *p* < 0.05; Figure 3C).

Concomitantly, intracellular ROS levels surged upon C12-HSL treatment, with donors exhibiting 1.50 ± 0.05-fold elevation (*p* < 0.0001) and recipients showing 1.27 ± 0.04-fold increase (*p* < 0.0001) via DCF fluorescence (Figure 3D). This oxidative burst correlated with transcriptional upregulation of SOS response genes (*recA*, *lexA*), mechanistically linking membrane disruption and ROS-driven genetic plasticity to enhanced conjugative efficiency.

### 3.4. C12-HSL Transcriptional Reprogramming Optimizes Membrane Architecture and Genetic Plasticity for Conjugation

**Figure 4:**
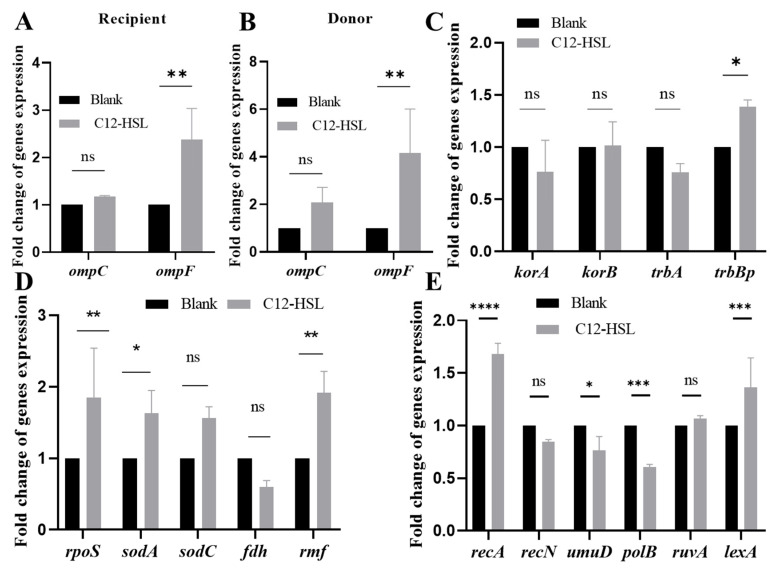
Transcriptional reprogramming induced by C12-HSL in genes governing membrane architecture, plasmid transfer, and cellular stress adaptation. (**A**) Quantitative PCR analysis of membrane-associated genes (*ompC*, *ompF*) in recipient *E. coli* strains treated with 200 μM C12-HSL. mRNA levels quantified using SYBR Green chemistry with 16S rRNA normalization; fold-changes calculated relative to untreated controls. (**B**) Identical qPCR assessment of membrane-associated genes (*ompF*, *ompC*) in donor strains. (**C**) Expression profiling of conjugation machinery genes (*korA*, *korB*, *trbA*, *trbBp*). Total RNA was extracted with Trizol reagent, reverse transcribed using HiScript III Reverse Transcriptase and amplified via SYBR Green qPCR. (**D**) Quantification of oxidative stress-responsive genes (*rpoS*, *sodA*, *sodC*, *fdh*, *rmf*) using identical qPCR methodology. (**E**) Analysis of SOS response pathway genes (*lexA*, *recA*, *recN*, *polB*, *ruvA*, *umuD*) under identical experimental conditions as (**C**). Error bars indicate standard deviation (*n* = 3 biological replicates). Statistical significance: * *p* < 0.05, ** *p* < 0.01, *** *p* < 0.001, **** *p* < 0.0001, ns (non-significant).

Comprehensive qPCR analyses (16S rRNA-normalized) delineated C12-HSL-driven transcriptional rewiring in *E. coli*. Both donor and recipient strains exhibited significant *ompF* upregulation (donors: 4-fold, *p* < 0.01; recipients: 2.38-fold, *p* < 0.01), whereas *ompC* remained unaffected (Figure 4A,B). Conjugation efficiency was further potentiated by specific induction of *trbBp* (1.38-fold, *p* < 0.05; Figure 4C), encoding a critical conjugative pore component.

Concurrently, oxidative stress defense genes were selectively activated: *rpoS* (stress master regulator, 1.85-fold, *p* < 0.01), *sodA* (superoxide dismutase, 1.7-fold, *p* < 0.05), and *rmf* (ribosome modulation, 2-fold, *p* < 0.05; Figure 4D). SOS response elements *recA* (recombinase, 1.68-fold, *p* < 0.001) and *lexA* (repressor cleavage, 1.37-fold, *p* < 0.001) were coordinately elevated (Figure 4E). This tripartite reprogramming—enhancing outer membrane permeability (*ompF* upregulation), conjugative pore assembly (*trbBp* upregulation), and DNA repair capacity (*recA-lexA* upregulation)—mechanistically enables accelerated antibiotic resistance transfer.

### 3.5. rmf and rpoS Govern Quorum Sensing-Driven Antibiotic Resistance Dissemination

**Figure 5:**
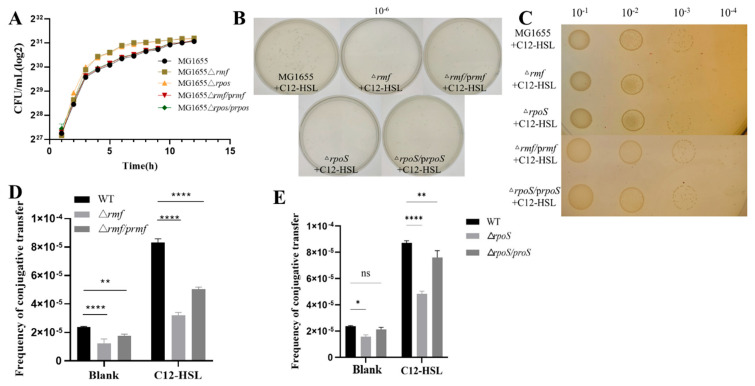
Analysis of bacterial growth and plasmid conjugation efficiency in genetic variants. (**A**) Growth kinetics of wild-type MG1655, isogenic deletion mutants (Δ*rmf*, Δ*rpoS*), and genetically complemented strains (Δ*rmf/prmf*, Δ*rpoS/prpos*). Bacterial growth was assessed by enumerating colony-forming units (CFU/mL, log_2_-transformed) at hourly intervals over 15 h in LB at 37 °C with orbital shaking (220 rpm). (**B**) Plate count analysis of transconjugants following conjugation. Donor and recipient cells were co-incubated for 24 h with 200 μM C12-HSL. Transconjugants were quantified after plating serial dilutions (10^−6^ dilution shown) on antibiotic-selective agar. Strain designations: wild-type (MG1655), *rmf* mutant (Δ*rmf*), *rmf*-complemented strain (Δ*rmf/prmf*), *rpoS* mutant (Δ*rpoS*), and *rpoS*-complemented strain (Δ*rpoS/prpoS*). (**C**) Conjugation efficiency assessed by spot assay. Ten-fold serial dilutions (10^−1^ to 10^−4^) of conjugation mixtures cultured under 200 μM C12-HSL for 24 h were spotted onto selection plates. Results shown represent the 10^−3^ dilution spot for each strain. (**D**) Quantitative conjugation frequencies in *rmf* genetic variants. Plasmid transfer was measured after 24 h incubation under control conditions (Blank) or with 200 μM C12-HSL. Evaluated strains: wild-type (WT), *rmf* deletion mutant (Δ*rmf*), and *rmf*-complemented derivative (Δ*rmf/prmf*). (**E**) Conjugation frequencies in *rpoS* genetic variants. Experimental conditions identical to panel (**D**). Strains analyzed: wild-type (WT), *rpoS* deletion mutant (Δ*rpoS*), and *rpoS*-complemented derivative (Δ*rpoS/prpoS*). Error bars indicate standard deviation (*n* = 3 biological replicates). Statistical significance: * *p* < 0.05, ** *p* < 0.01, **** *p* < 0.0001, ns (non-significant).

Comprehensive genetic dissection demonstrated that *rmf* and *rpoS* deletion mutants maintained near-wild-type growth kinetics (Figure 5A), enabling unambiguous attribution of conjugation defects to targeted gene loss under 200 μM C12-HSL. In visual assays, colony-forming unit (CFU) counts (Figure 5B) and spot plating (Figure 5C) revealed severe attenuation in Δ*rmf* (55.8% reduction, *p* < 0.001) and Δ*rpoS* (65.5% reduction, *p* < 0.0001) strains, with complementations achieving partial but significant restoration. In quantitative profiling assays, baseline conjugation frequencies were significantly impaired in Δ*rmf* (46% of WT) and Δ*rpoS* (35% of WT; *p* < 0.001). C12-HSL failed to hyperstimulate mutants, while complemented strains exhibited dose-dependent phenotypic rescue (Δ*rmf/prmf*: 75% recovery; Δ*rpoS/prpoS*: 60% recovery; *p* < 0.01 vs. mutants; Figure 5D,E).

This triad of evidence—growth-normalized phenotypes, visual suppression, and quantitative frequency deficits—definitively positions *rmf* (ribosome hibernation promoter) and *rpoS* (global stress regulator) as non-redundant amplifiers of AHL-driven conjugative transfer.

### 3.6. Genetic Control of Membrane Integrity and Oxidative Stress by rmf and rpoS

**Figure 6:**
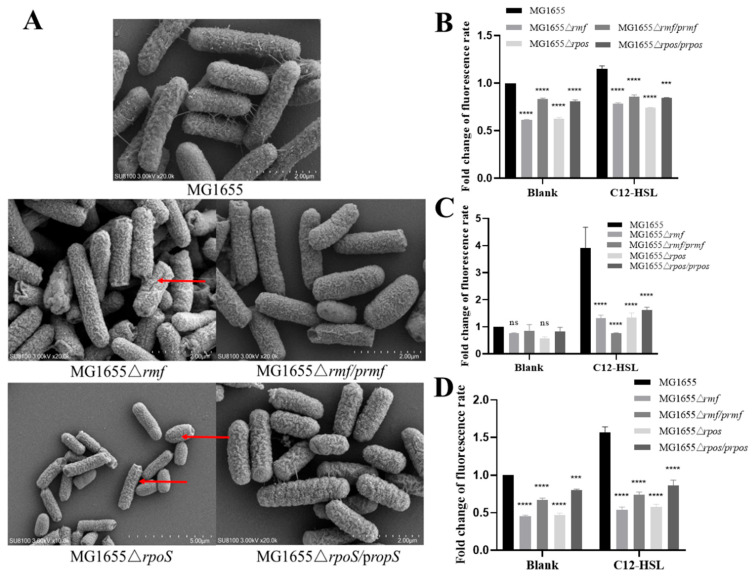
Phenotypic characterization of membrane integrity and oxidative stress responses in *rmf*- and *rpoS*-deficient strains. (**A**) Representative scanning electron micrographs depicting cellular surface integrity. Bacterial cultures (wild-type MG1655, deletion mutants Δ*rmf* and Δ*rpoS*, complemented strains Δ*rmf/prmf* and Δ*rpoS/prpoS*) were grown to mid-log phase in lysogeny broth at 37 °C, fixed with 2.5% glutaraldehyde, and imaged at 10,000× magnification. The red arrow indicates the damaged area on the bacterial surface. (**B**) Quantitative analysis of inner membrane permeability via PI exclusion assay. Bacterial suspensions were incubated in control buffer or 200 μM C12-HSL-supplemented buffer for 1 h. PI fluorescence intensity (λ_ex_/λ_em_ = 535/617 nm) was quantified. Strains shown correspond to Panel (**A**). (**C**) Determination of outer membrane permeability using NPN uptake assay. Fluorescence measurements (λex/λem = 350/420 nm) were performed after 30 min treatments paralleling Panel (**B**). (**D**) Intracellular ROS quantification with DCFH-DA fluorescence. Bacterial suspensions were loaded with DCFH-DA under indicated conditions, with fluorescence expressed relative to unstained controls. Error bars indicate standard deviation (*n* = 3 biological replicates). Statistical significance: ns (non-significant), *** *p* < 0.001, **** *p* < 0.0001.

Comprehensive characterization of *rmf* and *rpoS* mutants revealed their essential roles in maintaining structural and functional membrane integrity. High-resolution SEM imaging demonstrated substantial surface damage in Δ*rmf* and Δ*rpoS* mutants (Figure 6A), which was rescued in complemented strains. Quantitative permeability assays showed, in the inner membrane, Δ*rmf* and Δ*rpoS* exhibited 38% (*p* < 0.0001) and 37% (*p* < 0.0001) reduced PI uptake versus wild-type (WT) under basal conditions. While C12-AHL increased permeability in mutants, values remained significantly below WT levels. Complementations partially restored phenotypes (Figure 6B). In the outer membrane, NPN uptake decreased by 64% (Δ*rmf*) and 65.5% (Δ*rpoS*) post-C12-AHL exposure versus untreated WT (*p* < 0.0001), with partial recovery in complemented strains (Figure 6C). In oxidative stress, basal ROS levels were suppressed by 54.2% (Δ*rmf*) and 52.9% (Δ*rpoS*) versus WT (*p* < 0.0001). C12-AHL failed to fully induce ROS in mutants (Figure 6D).

**Figure 7:**
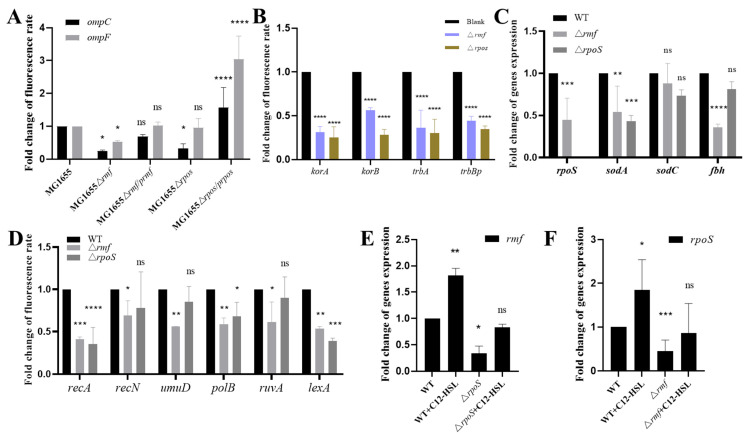
Comprehensive transcriptional analysis of stress response and conjugation-associated genes in *rmf/rpoS*-modulated strains. (**A**) Expression profiling of outer membrane porin genes (*ompC*, *ompF*). mRNA levels were quantified by SYBR Green-based qPCR in mid-log phase cultures of WT MG1655, deletion mutants (Δ*rmf*, Δ*rpoS*), and genetically complemented derivatives (Δ*rmf/prmf*, Δ*rpoS/prpoS*). Total RNA was extracted using TRIzol reagent. (**B**) Expression assessment of plasmid conjugation machinery genes. Transcription of *trbBp*, *korA*, *korB*, and *trbA* was measured via fluorescence qPCR in Δ*rmf* and Δ*rpoS* mutants relative to WT under uninduced conditions. cDNA synthesis employed random hexamer primers. (**C**) Oxidative stress gene quantification. mRNA fold changes for *rpoS*, *sodA*, *sodC*, and *fbh* were determined in wild-type, Δ*rmf*, and Δ*rpoS* strains with 16S rRNA normalization. (**D**) Transcriptional analysis of SOS response genes. Expression of *recA*, *recN*, *umuD*, *polB*, *ruvA*, and *lexA* was evaluated in WT and deletion mutants. (**E**) Regulatory impact of *rpoS* on *rmf* transcription. *rmf* mRNA levels were compared across WT and Δ*rpoS* strains incubated with/without 200 μM C12-AHL. (**F**) *rmf*-dependent control of *rpoS* expression. *rpoS* transcription was quantified via fluorescence qPCR in WT and Δ*rmf* strains incubated with/without 200 μM C12-AHL. Error bars indicate standard deviation (*n* = 3 biological replicates). Statistical significance: * *p* < 0.05, ** *p* < 0.01, *** *p* < 0.001, **** *p* < 0.0001, ns (non-significant).

To elucidate the regulatory functions of *rmf* and *rpoS* in *E. coli*, we quantified gene expression across key pathways. Membrane-associated gene analysis (Figure 7A) revealed significantly reduced *ompC* expression in both *Δrmf* and *ΔrpoS* mutants relative to WT (*p* < 0.0001), with *ompF* also downregulated (*p* < 0.05 in specific comparisons); partial restoration was observed in complemented strains. For plasmid conjugation genes (Figure 7B), *trbBp* expression decreased by 55.8% in *Δrmf* (*p* < 0.0001) and 65.5% in *ΔrpoS* (*p* < 0.001) versus the control, with all assessed genes exhibiting statistically significant suppression. Oxidative stress responses (Figure 7C) showed a 55.3% reduction in *rpoS* expression in *Δrmf* (*p* < 0.001), while *sodA* declined by 45.9% (*Δrmf*, *p* < 0.01) and 57.1% (*ΔrpoS*, *p* < 0.001); *sodC* and *fbh* remained unaltered. SOS response assays (Figure 7D) demonstrated pronounced decreases. *recA* expression was suppressed by 58.9% (*Δrmf*, *p* < 0.001) and 64.7% (*ΔrpoS*, *p* < 0.0001), and *lexA* expression was suppressed by 46.7% (*Δrmf*, *p* < 0.01) and 61% (*ΔrpoS*, *p* < 0.001). Transcriptional crosstalk studies (Figure 7E,F) indicated that *rpoS* deletion reduced *rmf* expression by 65.9% (*p* < 0.05), partially reversible by C12-AHL stimulation. Conversely, *rmf* ablation diminished *rpoS* transcription by 55.3% (*p* < 0.05) and impaired AHL-mediated induction.

## 4. Discussion

Our findings establish a hierarchical molecular mechanism through which the QS signal C12-HSL enhances conjugative plasmid transfer in *E. coli*. Central to this mechanism is the demonstration that exogenously supplied C12-HSL increases antibiotic resistance plasmid transfer frequency by up to 7.7-fold via a coordinated process involving membrane destabilization, oxidative stress induction, and, critically, reciprocal crosstalk between the master stress regulator RpoS and the ribosomal hibernation factor RMF. This hyperconjugation was effectively suppressed (95% reduction) by sub-inhibitory vanillin, confirming the dependence on specific AHL-mediated QS signaling. Genetic deletion of either *rpoS* or *rmf* profoundly attenuated the cellular response to C12-HSL, markedly decreasing conjugation frequencies (by 65.5% and 55.8%, respectively) and correspondingly diminishing membrane permeability changes, ROS accumulation, and transcriptional activation of key conjugation (*trbBp*) and stress adaptation (*sodA*, *recA*, *lexA*) genes. This combined genetic evidence definitively identifies the RpoS–RMF signaling node as a non-redundant hub essential for translating C12-HSL perception into a cellular state conducive to efficient DNA transfer.

The observation that C12-HSL enhances conjugation efficiency aligns with studies demonstrating AHL modulation of horizontal gene transfer in pathogens like *Pseudomonas aeruginosa* and *Salmonella enterica* [1,6,14,40]. However, the specific molecular components and integrated signaling hierarchy uncovered here contrast with certain established models. For instance, while research in *P. aeruginosa* has suggested AHL-promoted conjugation independent of ROS generation in specific contexts [41], our data in *E. coli* MG1655 reveal a strong mechanistic link: C12-HSL induced significant ROS accumulation (1.5-fold) and concurrently activated SOS-response genes, thereby connecting oxidative DNA damage signaling directly to enhanced conjugation. This divergence potentially reflects fundamental species- or system-specific variations in downstream QS circuitry. Furthermore, although the role of stress regulators like RpoS in bacterial adaptation, including potential effects on HGT, is acknowledged, our identification of RMF—a factor traditionally associated with translational silencing during nutrient stress—as an indispensable co-regulator with RpoS in mediating C12-HSL responsiveness constitutes a significant extension [42,43,44]. The substantial mutual transcriptional suppression observed in mutants (*rpoS* deletion reduced *rmf* expression by 65.9%; *rmf* deletion decreased *rpoS* expression by 55.3%), coupled with the strikingly similar attenuation phenotypes across all functional assays, suggests a level of functional interdependence and co-regulation between ribosomal dormancy (RMF) and global stress management (RpoS) previously unrecognized in HGT regulation. This model offers a cohesive explanation for prior associations [1,6,15,41] linking stationary phase/stress conditions to increased gene exchange, specifically identifying the RpoS–Rmf interaction as a critical integrative signaling hub.

These findings possess substantial implications for efforts to combat the global crisis of antibiotic resistance. By delineating a specific signaling cascade—initiated by a defined QS molecule (C12-HSL), transduced via increased membrane permeability (primarily through *ompF* upregulation) and oxidative stress signaling, and integrated by RpoS–RMF crosstalk to drive expression of conjugation machinery (*trbBp*) and DNA repair capacity—this study provides tangible targets for intervention. Strategies designed to disrupt this pathway hold considerable promise. Quorum quenching, validated here by vanillin’s potent blockade of C12-HSL action, emerges as a viable strategy to specifically inhibit resistance spread, particularly relevant in contexts like agriculture or wastewater treatment, where preventing dissemination without bactericidal activity is desirable. Moreover, the essentiality of the RpoS–RMF crosstalk reveals novel potential targets, such as agents disrupting this regulatory interaction or modulating ribosome hibernation, for specifically curbing QS-driven horizontal transfer of resistance genes without exerting broad selective pressure.

Despite establishing a robust mechanistic framework within the studied model system, several limitations merit consideration. Primarily, our conclusions derive from experiments using the *E. coli* K-12 strain MG1655 and the RP4-7 plasmid. The generalizability of the RpoS–RMF dependent mechanism across diverse *E. coli* lineages (e.g., pathogenic ST131) or other Gram-negative species harboring distinct conjugative plasmids requires empirical validation. Clinical and environmental isolates often possess additional regulatory complexities that might modulate this core pathway. Secondly, the experimental conditions were highly controlled laboratory settings in vitro. The dynamics of natural microbial communities—involving multi-species interactions, fluctuating nutrient levels, concurrent stresses (including sub-inhibitory antibiotics), and spatially structured environments like biofilms—could substantially influence the relative contribution of the identified C12-HSL > Membrane > ROS > RpoS–RMF > Conjugation pathway compared to observations in monoculture. Finally, while genetic complementation partially rescued mutant phenotypes (60–75% recovery), the incomplete restoration suggests either intrinsic limitations of plasmid-based gene expression in trans or the involvement of supplementary regulatory factors within this pathway awaiting discovery.

Future research should prioritize enhancing both the scope and ecological relevance of this work. Key avenues include (1) assessing the function of RpoS–RMF crosstalk in mediating C12-HSL effects across a diverse range of clinically relevant *E. coli* isolates and other significant Gram-negative pathogens (e.g., *Klebsiella pneumoniae*, *Acinetobacter baumannii*); (2) elucidating the molecular nature of the RpoS–RMF interaction—whether involving direct protein binding, transcriptional co-regulation, or intermediate signaling molecules—through techniques like ChIP-seq and co-immunoprecipitation; (3) evaluating the in vivo efficacy of disrupting this pathway, using QSIs like vanillin or specific inhibitors targeting the RpoS–RMF interface, within relevant animal infection models or complex microbial communities (e.g., gut microbiome simulators) to determine impact under physiologically realistic conditions; (4) investigating the interactions between sub-minimal inhibitory concentrations of clinically relevant antibiotics and QS-mediated conjugation more rigorously, particularly whether they potentiate, antagonize, or act independently of the C12-HSL pathway; and (5) exploring whether analogous signaling hubs, potentially involving different sigma factors or ribosome-associated proteins, mediate QS-dependent conjugation in Gram-positive bacteria.

In summary, this research significantly advances our comprehension of how QS directs a critical mechanism of antibiotic resistance dissemination. We have delineated a specific pathway: C12-HSL-mediated signaling enhances conjugative plasmid transfer in *E. coli* MG1655 through a coordinated sequence of increased membrane permeability (primarily via *ompF* upregulation), induction of oxidative stress leading to SOS DNA damage response activation, and, pivotally, the synergistic interplay between the RpoS sigma factor and the RMF ribosome hibernation factor. The reciprocal dependency between RpoS and RMF constitutes a novel and essential regulatory axis for translating extracellular QS signals into cellular changes that promote DNA exchange. Critically, from a public health perspective, this mechanistic insight identifies actionable targets, the C12-HSL/RpoS–RMF signaling axis, for disrupting a key driver of resistance gene spread in bacterial populations. Interventions designed to block this pathway (e.g., QS inhibitors targeting AHLs or RpoS/RMF function) hold potential to reduce the horizontal dissemination of high-risk resistance plasmids within clinical, agricultural, and environmental settings, thereby mitigating a major contributor to the global antibiotic resistance crisis. Although further validation in clinically and environmentally relevant contexts is warranted, these findings robustly position QS, particularly the activity of long-chain AHLs and the RpoS–RMF signaling nexus, as promising mechanistic targets for developing strategies to mitigate the spread of antibiotic resistance genes.

## 5. Conclusions

This study establishes that C12-HSL drives a hierarchical signaling cascade amplifying antibiotic resistance plasmid transfer by 7.7-fold (*p* < 0.001). This effect is mediated through concerted membrane destabilization via OmpF upregulation (4-fold; *p* < 0.01), oxidative stress-triggered SOS response (ROS: 1.5-fold, *recA*: 1.68-fold; both *p* < 0.001), and reciprocally dependent RpoS–RMF crosstalk. Deletion of *rpoS* or *rmf* attenuated conjugation (65.5% and 55.8%, respectively; *p* < 0.001), membrane permeability (NPN influx ≤65.5%; *p* < 0.0001), and stress gene induction. Critically, RpoS–RMF mutual transcriptional suppression (≤65.9%; *p* < 0.05) defines this hub as a non-redundant lynchpin for AHL-hyperconjugation. Our findings identify specific targets—QS signaling, membrane architecture, and RpoS–RMF interplay—to mitigate resistance spread.

## Data Availability

The original contributions presented in this study are included in the article/Supplementary Material. Further inquiries can be directed to the corresponding authors.

## Supplementary Materials

The following supporting information can be downloaded at: https://www.mdpi.com/article/10.3390/microorganisms13081837/s1, Table S1: Strains and plasmids used in this study; Table S2: Primers used in this study; Figure S1: Identification of primary and secondary recombinant bacteria by PCR.
