# Supplementary material for: Quorum-Sensing C12-HSL Drives Antibiotic Resistance Plasmid Transfer via Membrane Remodeling, Oxidative Stress, and RpoS-RMF Crosstalk
Source: Microorganisms. 2025 Aug 6;13(8):1837. doi: 10.3390/microorganisms13081837 (PMC12388090; doi:10.3390/microorganisms13081837)
Supplement: Supplementary file 1 [file microorganisms-13-01837-s001.zip › microorganisms-3771018-supplementary.pdf]

microorganisms

MDPI

Supplementary Materials

Table S1 Strains and plasmids used in this study

Strains and plasmids	Details	Reference
**Strains**		
MG1655	ATCC 47076	Purchased from NuoheBio (Yangzhou, China)
MG1655 Δ*rmf*	*rmf* knockout strain	Constructed in this study
MG1655 Δ*rmf*/ p*rmf*	*rmf* knockout strain complemented with p*rmf*	Constructed in this study
MG1655 Δ*rpoS*	*rpoS* knockout strain	Constructed previously and preserved in our laboratory
MG1655 Δ*rpoS*/p*rpoS*	*rpoS* knockout strain complemented with *rpoS*	Constructed previously and preserved in our laboratory
**Plasmids**		
pBR322	Expression vector, Amp^r	Takara Ltd.
RP4-7	Confer resistance to chloramphenicol and ampicillin	This plasmid was a kind gift from Prof. Xia Xiao, College of Veterinary Medicine, Yangzhou University.
EC600	Confer rifampicin resistance	This plasmid was a kind gift from Prof. Xia Xiao, College of Veterinary Medicine, Yangzhou
pKD3	Cm^r; Cm cassette teplate	Preserved in our laboratory
pKD46	Amp^r, λRed recombinase	Preserved in our laboratory
pCP20	Amp^r,Cm^r; Flp recombinase	Preserved in our laboratory

 https://doi.org/10.3390/microorganisms13081837

Table S2 Primers used in this study

Primers	Sequencing (5'-3')
ompC-F	AAGTAGTAGGTAGCACCAACATCA
ompC-R	GGGCGAACAAAGCACAGAA
ompF-F	GGTCTGCGTCCGTCCAT
ompF-R	GGTTGCGCCCACTTCA
korA-F	TCGGGCAAGTTCTTGTCC
korA-R	GCAGCAGACCATCGAGATA
korB-F	CTGGTCGGCTTCGTTGTA
korB-R	TGAAGTCACCCATTTCGGT
trbA-F	TGGAAACTCCCCTACCTCTT
trbA-R	CCACACTGATGCGTTCGTAT
trbBp-F	CGCGGTCGCCATCTTCACG
trbBp-R	TGCCCGAGCCAGTACCGCCAATG
rmf-F	ATGAAGAGACAAAAACGAGAT
rmf-R	TCAGGCCATTACTACCCT
rpoS-F	TTTTACCACCAGACGCAAGT
rpoS-R	GGAACTGTTATCGCAGGGAG
sodA-F	GAAAGGCGATAAACTGGCGG
sodA-R	GCGCCAGAAATAGCTTCACC
sodC-F	GCGCCTCGTCTGAAATCACT
sodC-R	TTACACCACAGGCATAGCGT
fdh-F	TACTGTCAACGGTGCGTGAA
fdh-R	TGATTTGTGCGTAGCCAGGT
lexA-F	GGAAGAGGAAGAAGGGTTGC
lexA-R	TCGGCTTGAATAAGGAAGGA
recA-F	CTACGCCTCTGTTCGTCTCG
recA-R	TTTGTTCTTCACCACTTTCACG
recN-F	TGTAGGGATTAGCGGTCCAA
recN-R	CACTTGTGGCAGGTGGGTAA
polB-F	TTCTGATGCCAACCATTCG
polB-R	GCTCCTGCTGAAACTGCTG
ruvA-F	AATGTCAGCGCAGCAGTTC
ruvA-R	TTTCAACAATCAAGCGTTCG
umuD-F	GCCCGACGGTACAGCTTATT
umuD-R	ACACCAAAGACATCCAGCGT
16sRNA-F	CCTACGGGAGGCAGCAG
16sRNA-R	ATTACCGCGGCTGCTGG
rmf-P1	ATGAAGAGACAAAAACGAGAT
rmf-P2	TCAGGCCATTACTACCCT
rmf-P3	ATGAAGAGACAAAAACGAGATCGCCTGGAACGGGCA<br>CATCAACGTGGTTATGTGTAGGCTGGAGCTGCTTCG
rmf-P4	GCGTCTGATAGGGACACATTTCTTTTGAGCGTCCGGC<br>GATGCCGGCCTGACATATGAATACTCCTTAG
rpoS-P1	AGGAACTGTTATCGCAGGGAG
rpoS-P2	CGTCATCTTGCGTGGTATCTT
rpoS-P3	CGGACCGTTCTCTTTTTAATCGGCCAGGATGTC<br>CAGCAACGCTTGTGTAGGCTGGAGCTGCTTCG
rpoS-P4	AGGAACTGTTATCGCAGGGAGCCACACAGCGT<br>GTGTTGGACGCCATATGAATACTCCTTAG
rmf-MC-F	CGCGGATCCATGAAGAGACAAAAACGAGATC
rmf-MC-R	CAGGTCGACTCAGGCCACTACCCTGT

rpoS-MC-F	GACGCTAGCATGAGTCAGAATACGCTGAAAGTT
rpoS-MC-R	GATGGATCCTTACTCGCGGAACAGCGC
prmf- F	GAAGAGACAAAAACGAGATC
prmf- R	CGTGCAAGATTCCGAATAC
prpoS- F	ACAGCATCGCCAGTCACTAT
prpoS- R	GATGCGTCCGGCGTAGAG

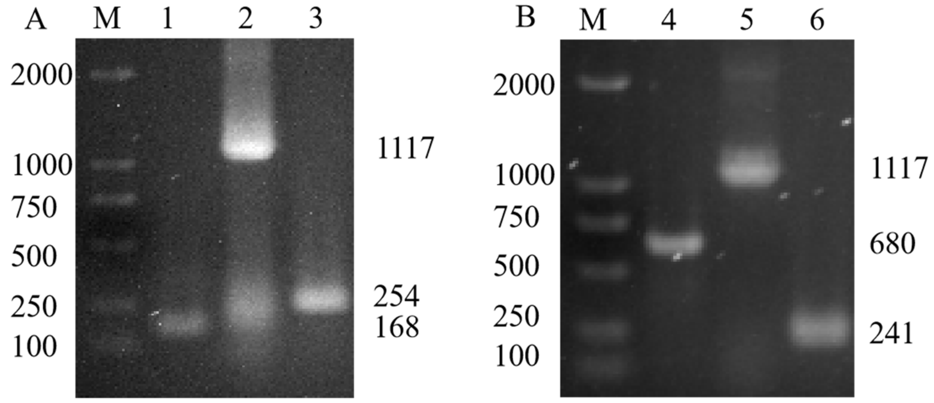

Figure S1. Identification of primary and secondary recombinant bacteria by PCR. Fig. S1 depicts the PCR-based confirmation of recombinant *E. coli* strains constructed through the λ-Red homologous recombination system. Part **A** shows the PCR analysis for the *rmf* gene knockout: lane M corresponds to the 2000 bp DNA marker; lane 1 represents MG1655 wild type; lane 2 indicates the primary recombinant strain MG1655△*rmf::cat*; and lane 3 designates the secondary recombinant strain MG1655△*rmf*. Part **B** exhibits the PCR analysis for the *rpoS* gene knockout: lane M corresponds to the 2000 bp DNA marker; lane 4 represents MG1655 wild type; lane 5 indicates the primary recombinant strain MG1655△*rpoS::cat*; and lane 6 designates the secondary recombinant strain MG1655△*rpoS*. Genomic DNA was extracted by boiling lysis, subjected to PCR amplification, and the products were separated by agarose gel electrophoresis on a 1% gel for visualization.
